# Autophagy and neurodegeneration: Unraveling the role of C9ORF72 in the regulation of autophagy and its relationship to ALS-FTD pathology

**DOI:** 10.3389/fncel.2023.1086895

**Published:** 2023-03-16

**Authors:** Rim Diab, Federica Pilotto, Smita Saxena

**Affiliations:** ^1^Department of Neurology, Center for Experimental Neurology, Inselspital University Hospital, Bern, Switzerland; ^2^Department for BioMedical Research (DBMR), University of Bern, Bern, Switzerland

**Keywords:** C9ORF72, ALS-FTD, autophagy, autophagy-lysosomal pathway, RAB proteins, endosomal trafficking, neurodegeneration

## Abstract

The proper functioning of the cell clearance machinery is critical for neuronal health within the central nervous system (CNS). In normal physiological conditions, the cell clearance machinery is actively involved in the elimination of misfolded and toxic proteins throughout the lifetime of an organism. The highly conserved and regulated pathway of autophagy is one of the important processes involved in preventing and neutralizing pathogenic buildup of toxic proteins that could eventually lead to the development of neurodegenerative diseases (NDs) such as Alzheimer’s disease or Amyotrophic lateral sclerosis (ALS). The most common genetic cause of ALS and frontotemporal dementia (FTD) is a hexanucleotide expansion consisting of GGGGCC (G4C2) repeats in the chromosome 9 open reading frame 72 gene (C9ORF72). These abnormally expanded repeats have been implicated in leading to three main modes of disease pathology: loss of function of the C9ORF72 protein, the generation of RNA foci, and the production of dipeptide repeat proteins (DPRs). In this review, we discuss the normal physiological role of C9ORF72 in the autophagy-lysosome pathway (ALP), and present recent research deciphering how dysfunction of the ALP synergizes with C9ORF72 haploinsufficiency, which together with the gain of toxic mechanisms involving hexanucleotide repeat expansions and DPRs, drive the disease process. This review delves further into the interactions of C9ORF72 with RAB proteins involved in endosomal/lysosomal trafficking, and their role in regulating various steps in autophagy and lysosomal pathways. Lastly, the review aims to provide a framework for further investigations of neuronal autophagy in C9ORF72-linked ALS-FTD as well as other neurodegenerative diseases.

## Introduction

The most common attribute of a plethora of neurodegenerative diseases (NDs) is the misfolding and aggregation of proteins, leading to the loss of certain populations of neurons within defined regions of the central nervous system (CNS). A hallmark of NDs is the formation of inter- and intracellular protein accumulations or aggregates in neuronal and glial cells, due to protein misfolding or unfolding (Meyer and Kaspar, 2017; Rahman et al., 2022). Autophagy is regarded as a major clearance pathway within cells, whereby damaged organelles and aggregated proteins are degraded. Autophagy involves the degradation of cytoplasmic contents, enclosed within double-membrane vesicles called autophagosomes, that are delivered to the lysosomes for destruction. It is very likely that small alterations in protein turnover or impaired protein homeostasis during one’s lifetime have cumulative effects that manifest NDs in aged individuals. This may be due to failure of cellular clearance machinery. Thus, autophagy has been systematically linked to the occurrence of pathological changes in several NDs such as in Alzheimer’s, Parkinson’s, and Huntington’s disease (Yue et al., 2009). In this review, we expand the relationship between impaired autophagy and NDs. Further, we provide deeper insights into the frequent genetic cause of amyotrophic lateral sclerosis (ALS) and frontal temporal dementia (FTD) due to the hexanucleotide repeat expansions (HRE) in the *C9ORF72* gene. Additionally, we discuss the impairments in the autophagy-lysosome pathway (ALP) that were recently identified in *C9ORF72*-ALS and FTD cases and in preclinical model systems. Moreover, we also highlight the physiological function of C9ORF72 protein in regulating and modulating the ALP machinery and cellular trafficking. Lastly, we touch upon some strategies to restore functional ALP, to ameliorate and restore the cell clearance machinery and to rescue the degeneration of neurons in ALS-FTD.

## Functional autophagy

Four forms of autophagy have been identified: macroautophagy, microautophagy, chaperone-mediated autophagy, and crinophagy (Csizmadia and Juhász, 2020). However, macroautophagy is the most well studied process and is generally referred to as autophagy. Autophagy is tightly regulated by metabolic signals, by reducing equivalents, and by nutrient status, and it is particularly sensitive to energy levels. (Ryter et al., 2019). Unc-51-like Kinase 1 (ULK1) is a crucial autophagy initiator. Mammalian target-of-rapamycin mTORC1, together with adenosine monophosphate activated protein kinase (AMPK), are responsible for ULK1 phosphorylation, where ULK1 and AMPK interact in a nutrient-dependent way (Shang et al., 2011). Nevertheless, in conditions of nutrient starvation, ULK1 is dephosphorylated specifically at Ser638 and Ser758 (Shang et al., 2011). ULK1 must be properly phosphorylated to associate with AMPK, as reported by Shang et al. (2011). The interaction with AMPK can be compromised by a single serine-to-alanine mutation (S758A) at ULK1. This mutant ULK1-S758A initiates starvation-induced autophagy at a faster rate than the wild-type ULK1, but has no effect on the maximal autophagic capacity as starvation lengthens. Autophagy commences with the formation of autophagosomes that sequester the cytoplasmic organelles/inclusions targeted for destruction. The initiation of autophagy, leads to the assembly of ATG proteins at a specialized site, known as the pre-autophagosomal structure (PAS) (Mizushima et al., 2011; Rubinsztein et al., 2012). PAS subsequently undergo synchronized fusion of membranes, derived from endosome, Golgi and plasma membranes, to form the phagophore (Rubinsztein et al., 2012). Autolysosomes are formed due to the fusion of autophagosomes to free lysosomes, leading to the breakdown of the inner autophagosomal membrane and the ingestion of cargo by lysosomal hydrolytic enzymes. The lysosomes may export the catabolites produced during autophagosome breakdown to the cytoplasm, where they can be recycled to create new macromolecules (Mahapatra et al., 2021).

## Autophagic lysosome reformation (ALR)

**FIGURE 1:**
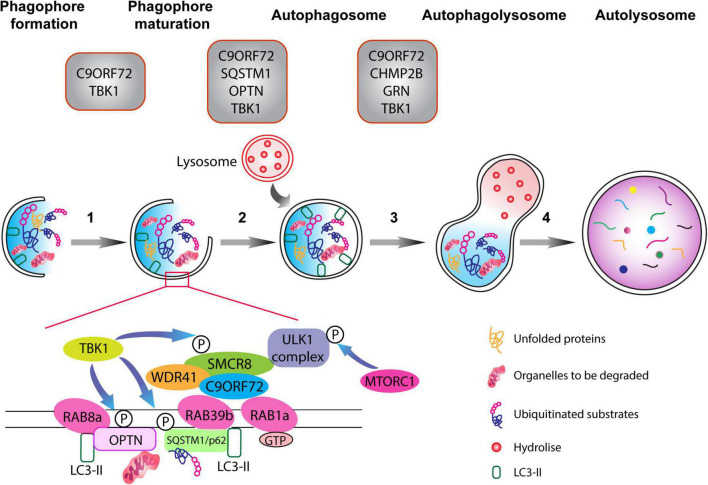
The autophagy pathway. Schematic representation of different phases of the autophagy pathway from phagophore formation to the degradation of dysfunctional organelles and ubiquitinated proteins (the autophagic cargo) within autolysosomes. Top: gray boxes depict the various proteins associated with ALS-FTD that normally function at defined stages of the autophagolysosome formation. Autophagy is composed of multiple steps (1) phagophore formation, where double membrane phagophore forms around damaged molecules, unfolded proteins, and compromised organelles, (2) phagophore maturation and elongation, (3) the formation of the mature phagophore carrying the autophagic cargo, (4) the fusion of the phagosome with lysosome containing hydrolytic enzymes, which degrade the content of the autophagic cargo. The C9ORF72-WDR1-SMCR8 complex regulates autophagy at the initiation and progression phases (steps 1–3). RAB1a GTPase can recruit the C9ORF72 complex to engage the ULK1 complex during the initial phase of the autophagy pathway. Importantly, RAB8a and RAB39b, respectively, bind to OPTN and SQSTM1/p62, and also interact with the C9ORF72-WDR1-SMCR8 complex. Finally, phosphorylation of SCMR8 by TBK1 is crucial for the initiation of the autophagy pathway.

Following the breakdown of autophagic substrates, the levels of amino acids in the cytoplasm rise and mTORC1 is activated (Chen X. et al., 2021). In addition to negatively regulating autophagy and preventing its overactivation, mTORC1 reactivation aids in the regeneration of lysosomes from autolysosomes (Figure 1). When this process, known as ALR, is compromised, the cells become far more vulnerable to starvation-induced cell death (Chen X. et al., 2021; Nanayakkara et al., 2022). Among all the different cells of our body, neurons are particularly vulnerable to compromised ALR, which predominantly impacts correct neuronal functioning. Unsurprisingly, ALR impairment is linked to an ever-increasing number of diseases, and classified as a subgroup of lysosomal disorders, involving an imbalance in lysosome homeostasis, leading to autophagy inhibition (Nanayakkara et al., 2022). Mutations in different genes, such as spastizin and spatacsin are associated with autosomal recessive hereditary spastic paraplegia. The loss of function of spatacsin in mice causes hereditary spastic paraplegia-like phenotypes with degeneration of cerebellar Purkinje cells and cortical neurons (Varga et al., 2015). Both spastizin and spatacsin are physiologically important components of the ALR. Loss of spastizin or spatacsin resulted in the compete loss of free lysosomes that are required to fuse with autophagosomes, leading to an accumulation of autolysosomes and the subsequent failure of ALR. Defects in the regulation of ALR might ultimately result in the decline of functional lysosomes, leading to reduced autophagic clearance. This decline results in the accumulation of non-degraded toxic proteinaceous and membranous material and, finally, neuronal death (Chang et al., 2014).

Likewise, ALR dysfunction is causally implicated in autosomal recessive spastic ataxia of Charlevoix-Saguenay (ARSACS), a fatal, neurodegenerative disorder, wherein cerebellar Purkinje cells selectively degenerate, leading to spasticity and ataxia (Bagaria et al., 2022; Francis et al., 2022). ARSACS is caused by mutations in the gene of sacsin molecular chaperone (SACS), leading to a loss of function phenotype. Normally, SACS binds to the motor-protein KIF5B, which is then involved in the extrusion of autolysosome membrane buds along microtubules to create reformation tubules during ALR (Du et al., 2016; Francis et al., 2022). Loss of SACS function inhibits the generation of reformation tubules at autolysosomes during ALR, (Francis et al., 2022). In conclusion, ALR plays an important role in maintaining cellular health, and the impairment of this process may contribute to the development of NDs. However, more research is needed to fully understand the exact mechanisms involved and how they could be targeted for the development of new therapies for these debilitating diseases.

## The cross talk between autophagy and UPS

Besides autophagy, it is vital to consider the impact of the ubiquitin-proteasome system (UPS) as one of the major quality control mechanisms involved in maintaining cellular homeostasis in eukaryotes. Both systems use ubiquitination as a degradation signal for their targets, but distinct processes are at work. Short-lived proteins and soluble misfolded proteins are degraded by UPS, whereas long-lived proteins, insoluble protein aggregates, whole organelles (such as mitochondria, peroxisomes), and intracellular parasites are destroyed by autophagy (Kocaturk and Gozuacik, 2018). There are links and reciprocal control mechanisms between both degradation pathways, in addition to an indirect connection between the two systems *via* ubiquitinated proteins (Nedelsky et al., 2008). The two degradative pathways play compensatory roles toward each other: when the function and ability of one process is mitigated or inhibited, the other’s activity levels rise dramatically to compensate for the deficits in the other pathway (Kocaturk and Gozuacik, 2018). The inhibition of the UPS using proteasome inhibitors (Wu et al., 2008; Selimovic et al., 2013; Fan et al., 2018) or by genetic manipulations (Demishtein et al., 2017) led to the induction of autophagy in cells as observed by the increased expression of autophagy-associated genes ATG5 and ATG7. This upregulation required the ER stress-dependent phosphorylation of eukaryotic translation initiation factor-2 alpha (eIF2α) (Zhu et al., 2010). Proteasome inhibition led to an increase in autophagy which correlated with the activation of two major regulators of autophagy, namely AMPK and mTORC1 (Xu et al., 2012; Jiang et al., 2015). Proteasome inhibition was also linked to an increase in sequestosome 1 (SQSTM1/p62) and GABARAPL1 expression by both Nrf1-dependent and independent pathways (Sha et al., 2018). Conversely, impairment in autophagy paralleled the activation of the UPS. Suppression of autophagy *via* chemical compounds, or *via* shRNA-mediated knock down of ATG genes, led to elevated activity of the UPS, mainly due to the increase in expression of proteasomal subunits (Lü and Wang, 2013). In another study, 3-MA-mediated autophagy inhibition in cultured neonatal rat ventricular myocytes increased chymotrypsin-like activity of proteasomes (Tannous et al., 2008).

The chaperone machinery, which oversees the quality control for proper protein folding, detects soluble portions of proteins with a folding issue and directs them to the UPS for destruction (Saibil, 2013). One of the E3 ligases that adds a K48-linked ubiquitin chain to unfolded or misfolded proteins has been identified as CHIP, a Hsp70 and Hsp90 chaperone interactor (Wang T. et al., 2020). The Hsp70 complex interacts with the BAG family of proteins, particularly BAG1, to initiate the proteasomal degradation of client proteins (Chen Y. et al., 2021). On the other side, aggresome production is necessary for the removal of insoluble aggregate-prone proteins. Ubiquitination *via* E3 ligases such as Parkin, CHIP, TRIM50, and HRD1 is the first step to priming insoluble protein aggregates (Olzmann et al., 2007; Olzmann and Chin, 2008; Mishra et al., 2009; Zhang and Qian, 2011). The process of aggresome formation is primarily achieved by HDAC6, serving as a linker protein between K63-ubiquitinated aggregates and dynein (Matthias et al., 2008). The aggregates are then transported toward microtubule organizing centers (MTOCs), resulting in their stacking up within the cytoplasm as aggresomes (Kopito, 2000). The direct interaction of SQSTM1/p62 and NBR1 with the ubiquitinated condensates further promotes their aggregation *via* their polymerization domain, followed by the delivery of ubiquitinated cargo to the autophagosomes (Ichimura et al., 2008; Lamark and Johansen, 2012). Alternatively, an autophagy-related protein known as ALFY has been identified as the main component in the onset of selective autophagy and the degradation of the aggresomal units (Clausen et al., 2010; Filimonenko et al., 2010).

## Relationship between autophagy and neurodegeneration

Protein aggregates or inclusion bodies are common hallmarks of age-related neurodegenerative disorders (Dugger and Dickson, 2017). The disruptive nature of these aggregates is characterized by their ability to confer toxicity, and consequently impair physiological neuronal functions, including axonal transport, synaptic integrity, mitochondrial bioenergetic machinery, and the regulation of transcription (Higuchi et al., 2002; Ruegsegger and Saxena, 2016; Dugger and Dickson, 2017; Batool et al., 2019). Several studies have shown how deregulation of autophagy associates with a high correlation to NDs like Alzheimer’s disease (AD) and Parkinson’s disease (PD). Moreover, recent studies have established a significant relationship between impaired autophagy and the onset of psychiatric disorders, such as schizophrenia and bipolar disorders (Merenlender-Wagner et al., 2014; Vanderplow et al., 2021). Current research has also provided novel insights into the intricate roles of autophagy in the regulation of lysosomal degradation, and the subsequent recycling of the lysosomal components, enabling the maintenance of an equilibrium between pro-survival and cell death responses (Bar-Yosef et al., 2019).

Due to impairments in the ALP, protein aggregation is often complementary to the decline in physiological degradative function of neurons. In the case of NDs, ALP dysfunction can be attributed to multiple factors, for example loss of function mutations that directly impact ALP functionality. Frequently, loss of function mutations are coupled with gain of toxic function of the genes that promote toxic protein aggregation, thereby contributing to the disruption of physiological cellular function and leading to neuronal death (Stavoe and Holzbaur, 2019; Monaco and Fraldi, 2020). CNS-specific knockdown of autophagy genes, *Atg5*, *Atg7*, and *FIP200*, led to mice exhibiting behavioral and motor deficits and dramatically reduced lifespan due to impaired autophagolysosome clearance, highlighting the crucial role of autophagy in maintaining neuronal homeostasis (Hara et al., 2006; Komatsu et al., 2006; Liang et al., 2020). Several misfolded proteins that are linked to NDs directly target the ALP, however, the question of whether ALP itself may be considered protective or destructive, or even as an active contributor to neuronal death remains unclear. Although ALP is highly complex in nature, nevertheless, it is well acknowledged that boosting it might have neuroprotective effects against neurodegeneration (Bossy et al., 2008). For example, boosting the ALP *via* the mTORC1 inhibitor rapamycin was shown to be neuroprotective against protein aggregates in *in vivo* models of Huntington’s disease (HD) (Ravikumar et al., 2004).

Some studies identified autophagy as a facilitator of apoptotic pathways, for instance *in vitro* assays on astrocytes have shown that autophagy can trigger the activation of caspase 8 *via* the degradation of caveolin-1 (Chen et al., 2018; Peng et al., 2019). A new form of cell death involving the autophagy pathway, called karyoptosis, has been proposed for the CNS. Karyoptosis is induced by the chronic inhibition of autophagy and characterized by cytoplasmic relocation of Lamin B1, together with LC3 and the SQSTM1/p62 receptor (Dou et al., 2015; Baron et al., 2017; Baron and Fanto, 2018). Karyoptosis, has been observed in models of AD and polyglutamine expansion (PolyQ) diseases where caspase-3 independent cell death has been detected (Turmaine et al., 2000; Yang et al., 2008). The mechanisms linking neuroprotective effects of autophagy, or those mediating cell death, are still debated. This highlights a more complex role for autophagy, not only in sustaining neuronal physiology, but also as an ever-present challenge for the treatment of NDs.

## Autophagy and autophagic lysosome reformation (ALR) impairments in NDs

Increasing evidence suggests that autophagy is implicated in neuronal disorders, ranging from AD and FTD, to movement disorders such as PD, ALS, multiple sclerosis and polyglutamine repeat diseases such as HD (Nah et al., 2015). As described in recent literature, dysfunctional autophagy is primarily implicated in the development of AD in human patients. The occurrence of impaired maturation or fusion of lysosomes with autophagosomes, or their movement toward the body of the neuronal cell, is commonly observed in AD neurons. Further, the presence of endosomal-lysosomal dysfunction has also been observed. In post-mortem human brain and in a mouse model of AD, this dysfunction is evident *via* the accumulation of double membrane autophagic vesicles (AVs) containing Aβ within dystrophic neurons and dendrites (Nixon et al., 2005; Boland et al., 2008). At the level of neuropathology, striking ultrastructural changes observed in AD involve the accumulation of AVs inside affected neurons (Terry et al., 1964; Nixon et al., 2005). Indeed, the extent of AVs accumulation in AD is so prominent that it often resembles AVs accumulation seen in lysosomal storage disorders (Nixon et al., 2008).

Recently, it was shown by Lee et al. (2022), that in AD, AVs are strongly immunoreactive to Aβ and are packed into large membrane blebs forming flower-like perikaryal rosettes. This unusual pattern of AV accumulation has been named as PANTHOS (poisonous anthos flower), and is widely observed within human post-mortem AD brains (Lee et al., 2022). Impairments in autophagic flux are known to occur in Aβ metabolism not only in the clearance and secretion process, but also in its generation (Turmaine et al., 2000). For instance, amyloid precursor protein (APP) and subunits of the γ-secretase complex are localized to autophagosomes, thus confirming a link between Aβ metabolism and autophagic flux (Di Meco et al., 2020). Rapamycin treatment of animal models of AD significantly improved cognitive skills. Additionally, reduced intracellular Aβ and extracellular amyloid deposition was observed, thus strongly linking AD with autophagy dysfunctions (Caccamo et al., 2010; Chandarlapaty et al., 2011). Moreover, brain specimens of AD patients were characterized by the presence of hyperphosphorylated tau colocalizing with LC3, an autophagosome marker, together with SQSTM1 (Piras et al., 2016).

In HD, a PolyQ expansion disorder, the gradual and progressive neuronal loss causes death of the striatal neurons. Compelling research points to autophagy as being crucial to the degradation of mutant Huntingtin (mHTT) protein (Martin et al., 2015). Indeed, research on HD animal models and human HD cell lines revealed a distinct autophagy-related deficit, whereby the AVs’ capacity to identify and capture cytosolic cargo is compromised (Martinez-Vicente et al., 2010). Of note, different post-translational events, such as SUMOylation, are known to play a role in autophagy in the context of HD. SUMOylation of the mHTT makes it more toxic to nerve cells. SUMO1 deletion in a humanized mouse model of HD, and in human fibroblasts, has been shown to boost and promote autophagic flux by increasing the association of mHTT with LAMP1 and LC3. This interaction also decreases the solubility of the toxic mHTT protein and its association with SQSTM1 (Ramírez-Jarquín et al., 2022). Additionally, a recent finding from Oh et al. (2022) reported that human fibroblasts directly reprogrammed to medium spiny neurons from HD patients show impairments in functional autophagy. Specifically, the authors identified an aging-associated upregulation of a microRNA (miR-29b-3p) that contributes to degeneration by targeting the human STAT3 3’ untranslated region, which is relevant for functional autophagy (You et al., 2015).

Parkinson’s disease is characterized by tremors, muscular rigidity, and bradykinesia caused by progressive loss of dopaminergic neurons in the substantia nigra pars compacta, followed by the accumulation of insoluble proteins in Lewy bodies (Gelb et al., 1999). It is interesting to note that various PD mutations can influence different autophagy phases. The pathophysiology of familial PD is caused by an accumulation of α-synuclein in Lewy bodies (Polymeropoulos et al., 1997; Krüger et al., 1998). It is assumed that wild-type α-synuclein, a protein with unidentified function, regulates dopamine neurotransmission at the pre-synaptic level and is routinely transported into lysosomes for chaperone-mediated autophagy (Winslow and Rubinsztein, 2011; Bar-Yosef et al., 2019). α-synuclein compromises ALP by directly inhibiting RAB1a, thereby impairing autophagy initiation. In addition, α-synuclein promotes the retention of TFEB, a key transcription factor regulating lysosomal biogenesis (Winslow et al., 2010; Settembre et al., 2011; Decressac et al., 2013). Interestingly, the process of ALR was impaired in PD neurons, leading to a deficit in functional lysosomes required to maintain prolonged autophagic clearance of α-synuclein. Notably, deficiency in glucosylceramidase beta 1 (GBA1) and cyclin G associated kinase (GAK) are established as genetic risk factors in 5–10% of cases of PD (Latourelle et al., 2009; Tysnes and Storstein, 2017; Avenali et al., 2020; Miyazaki et al., 2021). GBA1 deficiency specifically reduces lysosome function, leading to autophagy inhibition (Schöndorf et al., 2014; Brooker and Krainc, 2021; Kuo et al., 2022). GBA1 is required for ALR, and its deficiency leads to impaired lysosome homeostasis (Awad et al., 2015; Magalhaes et al., 2016). The neuronal accumulation of α-synuclein has been observed to coincide with ALR suppression, as during normal circumstances α-synuclein is cleared by autophagy (Magalhaes et al., 2016). Thus, ALR impairments might be causally associated with autophagy deficits and the accumulation of toxic α-synuclein species in the brain (Winslow et al., 2010; Magalhaes et al., 2016).

## Relationship between autophagy and ALS-FTD mutations

In the case of ALS and FTD a peculiar relationship exists, as ALS and FTD represent two extremes of an overlapping clinico-pathological disease spectrum. They not only manifest different intermediate phenotypes, but also exhibit several shared pathological aspects between the two diseases (Andersen, 2013). *C9ORF72* mutations are the most common genetic cause of ALS and, notably, most ALS-FTD causing monogenetic mutations overlap and mainly associate with endosomal-lysosomal and/or ALP. Point mutations in charged multivesicular body protein 2B (CHMP2B), valosin containing protein (VCP), ubiquilin 2 (UBQLN2), optineurin (OPTN), cyclin F (CCNF), TANK binding kinase 1 (TBK1), and sequestosome 1 (SQSTM1/p62), have been causally implicated in ALS-FTD pathology (Renton et al., 2014; Oakes et al., 2017; Rayner et al., 2021; Chua et al., 2022). Notably, systematic neuropathological analyses have revealed that 95–97% of patients with ALS and approximately 50% of patients with FTD share the presence of cytoplasmic TARDBP inclusions (Neumann et al., 2006; Hasegawa et al., 2008). In addition, there is co-accumulation of TARDB together with SQSTM1/p62, suggestive of impairments/disturbance in autophagy (Chua et al., 2022).

The fact that different mutations in proteins, which take part in the ALP, are causally liked to ALS-FTD clearly suggests that autophagy dysfunction is a key player in ALS-FTD disease progression. For instance, mutations in genes involved in the autophagic flux specifically target key molecular players in the ALP, altering their physiological function. For example, CHMP2B is part of the ESCRT-III complex that is involved in regulating endosomal sorting of transmembrane proteins as well as regulating the fusion of the lysosomal vesicles with the autophagosome (Krasniak and Ahmad, 2016). Numerous mutations in CHMP2B are associated with NDs, mainly involving the ALS-FTD spectrum. Within the ALS-FTD spectrum, a C to A substitution causing a T104N substitution, an A to G substitution resulting in a I29V substitution, and an A to G substitution leading to a Q206H substitution are all linked to primary muscular atrophy (Cox et al., 2010; Isaacs et al., 2011). Also, a G to T substitution leading to a D148Y mutation is associated with semantic dementia (Skibinski et al., 2005). Another ND, related to but not a part of the ALS-FTD spectrum, and caused by a CHMP2B mutation, is involved in corticobasal degeneration. This pathology is mainly due to an A to G substitution, resulting in a N143S mutation (van der Zee et al., 2008). Moreover, a dominant mutation in intron 5 of CHMP2B (CHMP2B*^Intron5^*) is linked to a fraction of heritable FTD associated with chromosome 3 (FTD-3) (Skibinski et al., 2005). Mouse models of CHMP2B*^Intron5^* have revealed a characteristic autophagy phenotype, with increased SQSTM1/p62 levels as well as prominent ubiquitin deposits throughout the brain (Ghazi-Noori et al., 2012; Gascon et al., 2014; Clayton et al., 2015). Notably, mutant neurons exhibited axonal swellings caused by the accumulation of membrane bound vessels arising from endolysosomal and autolysosomal phenotype (Ghazi-Noori et al., 2012).

It has recently been shown that ALS-FTD-linked mutations of TBK1 and SQSTM1 diminish the phosphorylation of SQSTM1, thereby compromising the binding and clearance of ubiquitinated cargo (Deng et al., 2020). During periods of oxidative stress, SQSTM1 mutation G427R inhibits SQSTM1 phosphorylation at Ser351, thereby impairing KEAP1-SQSTM1 association. This impairment leads to reduced NFE2L2/Nrf2-mediated gene expression and substantially enhanced stress granule formation (Deng et al., 2020; Lee D. H. et al., 2020). Importantly, in physiological conditions, SQSTM1/p62 plays a key role in directing the ubiquitinated substrates to the degradation machinery, either *via* autophagy or UPS. This involves the activation of ULK1, which phosphorylates SQSTM1/p62 enabling its increase in affinity for ubiquitinated substrates (Lim et al., 2015). In addition, SQSTM1/p62 directly interacts with the LC3, an autophagosome membrane protein leading to the sequestration of targets that need to be degraded *via* ALP (Liu et al., 2016). Another important interacting partner of SQSTM1/p62 is optineurin (OPTN), and together they form an autophagy complex receptor for the degradation of damaged mitochondria. Of note, different causative mutations of familial and sporadic ALS involve mutations and pathological variants of OPTN (Maruyama et al., 2010). Finally, another important player recently linked to ALS pathogenesis is TBK1, which phosphorylates SQSTM1/p62 at Ser403, thereby enabling its binding to ubiquitinated cargos. In pathological cases, TBK1 mutation causes impairments in the clearance of ubiquitinated substrates, therefore impairing autophagic flux (Matsumoto et al., 2015). Interestingly, preclinical studies have shown how the TBK1- SQSTM1/p62 axis is important for the clearance of TDP43 aggregates (Lee S. et al., 2020). The finding that most patients suffering from ALS-FTD do not harbor mutations in TARDBP gene, yet present cytoplasmic TARDBP inclusions, is indicative of disturbed TARDBP function, likely due to disturbances in the ALP or endosomal-lysosomal trafficking in ALS-FTD (Wood et al., 2021). Furthermore, the conditional knock out mouse model of TBK1 exhibits disrupted autophagy (Duan et al., 2019).

Point mutations in VCP are found in a fraction of patients suffering from either familial form of PD or ALS (Johnson et al., 2010). VCP plays an important role in autophagosome maturation and autolysosome formation under basal conditions, and in cells where proteasome is compromised, but not in cells undergoing starvation responses (Tresse et al., 2010). RNAi-mediated knockdown or overexpression of dominant-negative VCP led to the prominent accumulation of immature and abnormal autophagic vesicles. The expression of disease-related VCP mutants (R155H and A232E) cause autophagy deficits (Ju et al., 2009). These findings strongly associate deficits in ALP with the pathophysiology of ALS-FTD, highlighting specific neuronal vulnerability to dysregulated ALP in ALS-FTD.

## Physiological and pathological functions of C9ORF2 in autophagy pathways

**FIGURE 2:**
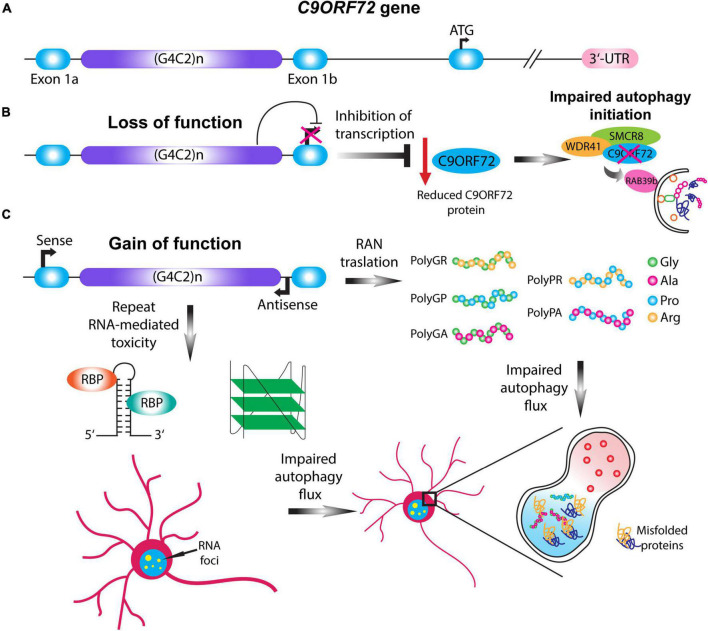
*C9ORF72* loss and gain of function mechanisms impair autophagy. **(A)** Schematic illustration of the *C9ORF72* gene. *C9ORF72*-ALS-FTD is characterized by a G4C2 hexanucleotide repeat expansion between exon 1a and exon 1b, which in patients can range up to thousands of repeats. **(B)** Haploinsufficiency of *C9ORF72* gene causes the loss of function of the C9ORF72 protein by transcription inhibition. Given its importance in the initial steps of phagophore formation, reduction in the C9ORF72 protein impairs autophagy initiation, destabilizing WDR41 and SMCR8 complex. **(C)** Two pathological mechanisms involving the gain of function of the HRE gives rise to RAN translation and repeat-linked RNA mediated toxicity. In the case of RAN translation, the HRE can be translated in a sense or antisense manner giving rise to five different dipeptide repeats proteins (DPRs): PolyGA, PolyGP, PolyGR, PolyPR, and PolyPA. In the case of RNA-mediated toxicity the translated HRE can form higher order structures that sequester different RNA binding proteins, and thus forming RNA foci. The gain of function mechanisms can impair the autophagic flux and the formation of the autolysosome, leading to the accumulation of misfolded proteins.

Hexanucleotide repeat expansions (G4C2) in the 5’ non-coding sequence of the *C9ORF72* gene was identified in 2011 as the most common inherited cause of ALS, FTD, and ALS-FTD (DeJesus-Hernandez et al., 2011; Renton et al., 2011). In contrast to healthy individuals which usually have 2 to 30 hexanucleotide repeats, ALS-FTD patients harbor 100 to even 1600 repeats (Buchman et al., 2013; Dols-Icardo et al., 2014). The pathogenic mechanisms by which HRE in the *C9ORF72* gene cause ALS are not fully understood, however, three distinct but mutually non-exclusive disease mechanisms have been proposed (Balendra and Isaacs, 2018; Schmitz et al., 2021): firstly, haploinsufficiency of the *C9ORF72* gene, leading to the loss of function associated with the reduced function of C9ORF72 protein (Shi et al., 2018); secondly, sequestration of RNA-binding proteins by RNA foci containing the *C9ORF72* HRE RNA (Haeusler et al., 2014, 2016); and lastly, repeat-associated non-AUG (RAN) translation of the repeat expansion, leading to the generation of dipeptide repeat proteins (DPRs) cause toxic gain of function in C9ORF72 ALS-FTD (Mori et al., 2013; Figure 2).

Although not much is known about the physiological functions of normal C9ORF72 protein, recent research has implicated an important role for C9ORF72 protein in autophagy. Structure-based modeling suggested that C9ORF72 protein, together with its binding partner SMCR8, belong to the Differentially Expressed in Normal and Neoplastic cells (DENN) domain subfamily of proteins. The DENN domain-containing protein family is characterized by a conserved N-terminal Longin domain and a C-terminal DENN domain (Zhang et al., 2012; Levine et al., 2013a,b; Jiang et al., 2022). The autophagic function of C9ORF72 was deduced from C9ORF72, or SMCR8 knockout mice, which exhibited dysregulation of mTORC1 or TFEB signaling (Ugolino et al., 2016; Shao et al., 2020). Three independent studies in 2016 established that the C9ORF72 protein forms a tripartite complex with SMCR8 and WDR41, and harbors guanine nucleotide exchange factor (GEF) activity, which it exerts upon small GTPases (Amick et al., 2016; Sellier et al., 2016; Sullivan et al., 2016; Yang et al., 2016). Notably, the tight interaction between C9ORF72 and SMCR8 is achieved *via* the DENN domain within the C9ORF72 protein (Yang et al., 2016). Importantly, C9ORF72 protein levels diminished when the expression of SMCR8 was inhibited (Zhang et al., 2018), while neither C9ORF72 nor SMCR8 knockout negatively influenced WDR41 protein levels (Ugolino et al., 2016). Similarly, loss of SMCR8 and WDR41 significantly reduced C9ORF72 protein levels and, conversely, SMCR8 levels were also decreased after C9ORF72 and WDR41 loss (Zhang et al., 2018; Lan et al., 2019). These studies indicate that a direct relationship exists between SMCR8 and C9ORF72 expression, likely *via* the stabilization of the two proteins, whereas WDR41 association might be involved in stabilizing the C9ORF72-SMCR8 heterodimers.

Furthermore, a systematic proteomic analysis of the human autophagy system, together with the functional analysis of selected genes within the autophagy pathway, identified SMCR8 as an interacting protein of FIP200. The latter is a protein involved in the ULK1 complex, and serves as the upstream component of the autophagy machinery (Behrends et al., 2010; Wong et al., 2013; Lin and Hurley, 2016). Moreover, the tripartite C9ORF72 complex interacts with the ULK1 complex *via* the C9ORF72 DENN domain, which also associates with SMCR8 and ATG13 (Tang, 2016; Webster et al., 2016; Cali et al., 2019; Ho et al., 2019). C9ORF72 localizes to lysosomal membranes and, during conditions of nutrient starvation, association between C9ORF72 and ULK1 complex is enhanced *via* an elevated interaction between SLC66A1/PQLC2 and WDR41 (Aoki et al., 2017; Beckers et al., 2021). The mechanistic aspects associated with the regulation of ULK1-linked autophagy initiation *via* C9ORF72 complex remain unclear. Both augmented transcriptional and post-transcriptional modifications have been implicated in this regulation (Yang et al., 2016; Amick et al., 2020). Nevertheless, several studies have conclusively established that the main effect of the partial loss of C9ORF72 function is reduced ULK1 activity and the subsequent reduction in autophagy (Sellier et al., 2016; Webster et al., 2016; Yang et al., 2016; Jung et al., 2017).

Further, based on these previous observations, the reduction in C9ORF72 protein level has been specifically linked to a detrimental effect on autophagy initiation (Ugolino et al., 2016; Shi et al., 2018; Wang M. et al., 2020). In neurons from *C9ORF72* depleted mice, both suppressed autophagy and reduced ULK1 protein levels were observed, suggesting that C9ORF72 and SMCR8 probably act against each other to regulate the autophagy initiation complex (Ho et al., 2019). Interestingly, ATXN2 with intermediate PolyQ repeats is associated not only with spinocerebellar ataxia type 2 (SCA2), but also with ALS (Elden et al., 2010), serving as a risk factor for *C9ORF72*-linked ALS (Zhang et al., 2017). Sellier et al. (2016) reported the existence of a distinct synergistic toxicity effect of ATXN2 intermediate PolyQ repeats, that induced motor neuron degeneration when coupled to *C9ORF72* haploinsufficiency. Importantly, C9ORF72 reduction partially affects neuronal survival by impairing initial steps in the autophagy pathway and therefore allowing the accumulation of ATXN2 aggregates. This results in neuronal death *in vitro* and in zebrafish models, where this synergistic toxic effect is associated with swimming impairments and compromised spinal axonal projections (Ciura et al., 2016; Sellier et al., 2016).

As the three pathological mechanisms of *C9ORF72*-ALS-FTD are mutually non-exclusive, several studies have investigated the synergy between these mechanisms in the disease pathology. Zhu et al. (2020) have shown that reducing or completely abolishing C9ORF72 protein expression inhibited the HRE-mediated increase in the induction of autophagy. This suppression of autophagy accelerated disease pathology by triggering the premature accumulation of DPRs, activating the glial cells and, ultimately, initiating hippocampal neuronal degeneration (Zhu et al., 2020). Yet another study has highlighted a similar synergy between the loss of C9ORF72 expression and DPRs accumulation. Bonvin et al. have shown that a double hit mechanism confers DPR toxicity *via* reduced autophagy due to diminished C9ORF72 protein levels. This reduction directly impairs functional autophagy promoting the toxic accumulation of DPRs such as PolyGA, PolyGR, and PolyGP. Furthermore, siRNA−mediated depletion of *C9ORF72* negatively impacted the recruitment of the SQSTM1/p62 autophagy adaptor protein to PolyGA aggregates, suggesting that C9ORF72 plays an important role at the first step of autophagosome formation (Boivin et al., 2020). In *C9orf72*-knockout mice, premature mortality is linked to dysfunctional immune responses, and they do not exhibit an overt neurodegenerative phenotype. However, upon crossing these knockout mice with mice carrying a repeat expansion transgene, the resulting toxic DPRs production attenuated autophagy induction. This attenuation led to the enhanced accumulation of DPRs, thus accelerating the pathology (Zhu et al., 2020). Based on these observations, one can envisage a toxic gain of function due to accumulations of DPRs, TARDBP, and HRE RNA; and, in parallel, the loss of function due to *C9ORF72* haploinsufficiency might lead to a vicious exacerbation of the disease pathology.

## Physiological and pathological functions of C9ORF72 in the regulation of RABs

The RAB family of proteins belongs to the RAS superfamily of proteins and, being enriched within distinct cellular organelles and membranes, plays a pivotal role in regulating membrane trafficking (Pang et al., 1988; Kiral et al., 2018). More than 60 RAB GTPases are present within the human genome, of which 24 are specific or highly enriched in expression within the CNS (Brighouse et al., 2010; D’Adamo et al., 2014). RAB proteins cycle between GTP-bound active and GDP-bound inactive states. The active vs. inactive state of RAB GTPases is tightly controlled by GDP-GTP exchange factors (GEFs) and GTPase-activating protein (GAPs). GAPs catalyze the hydrolysis of GTP to GDP, and GEFs promote the conversion of an inactive GDP-bound RAB to an active GTP-bound RAB (Barr and Lambright, 2010). Active RAB GTPases recruit downstream effector proteins in order to transmit signals involved in membrane trafficking and signaling. The effector proteins include tether proteins that facilitate vesicle docking and fusion, as well as GEFs and GAPs for other RAB GTPases (Grosshans et al., 2006). The active state and membrane association of RAB GTPases are further controlled by auxiliary proteins (Barr and Lambright, 2010; Chaineau et al., 2013; Ishida et al., 2016; Müller and Goody, 2018). The selective and highly defined membrane trafficking processes mediated by RAB GTPases rely on efficient interactions with other effector proteins such as motor proteins (kinesins and dyneins), tethering complexes (EEA1, Golgins, and the exocyst complex), coatamer complex (COPI, COPII, and clathrin), and SNAREs (Grosshans et al., 2006). Notably, the interaction of RABs with Guanosine nucleotide dissociation inhibitor (GDI), GEFs, GAPs and other effector proteins is regulated *via* the post-translational modifications of RABs such as phosphorylation (Pfeffer, 2013, 2017; Lamber et al., 2019).

**FIGURE 3:**
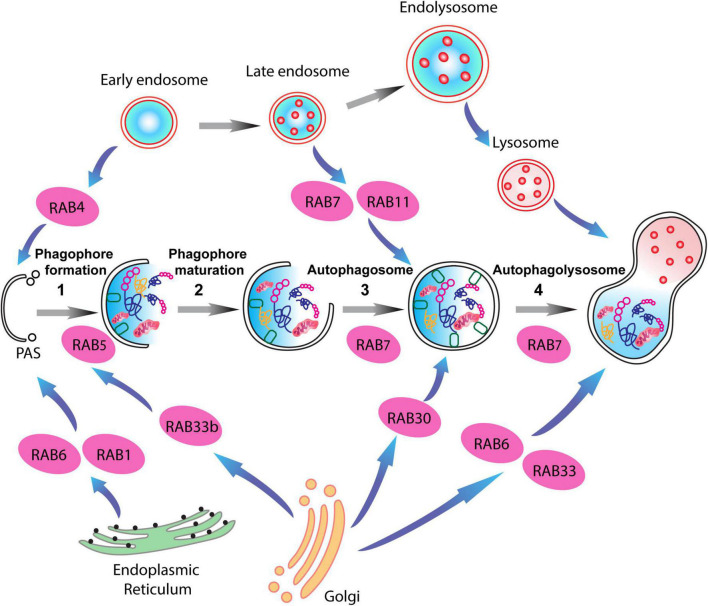
RAB proteins involved in different steps of the autophagy-lysosome pathway (ALP). Schematic representation of RAB GTPases and their binding partners in the ALP. RAB1, RAB6, and RAB4 are important for the formation of the pre-autophagosomal structure (PAS) providing membrane sources for the initiation of the autophagy pathway, while RAB5, RAB30, RAB33 are involved in autophagosome maturation together with RAB7, which is also involved in the fusion of the autophagosomes with late endosomes.

RABs are mainly located within different endosomes, where they systematically regulate different steps of endocytic trafficking independently or cooperatively. RAB4, RAB5, RAB7, RAB9, RAB10, RAB11, and RAB35 have been primarily associated with intracellular endocytic trafficking (Tsubokawa and Katayama, 1985; Zhang et al., 2022). RAB4 is predominantly located on early endosomes, and regulates the recycling of receptors from early endosomes directly back to the plasma membrane (fast recycling pathway) (Van Der Sluijs et al., 1991; Daro et al., 1996). In contrast, RAB11 is associated with the slow recycling pathway, and is localized in the recycling endosome, promoting the redistribution of receptors from the recycling endosome to the plasma membrane (Ullrich et al., 1996; Ren et al., 1998). RAB5 plays an important role in regulating vesicle transport from the plasma membrane to the early endosome by fostering the fusion of newly formed endocytic vesicles with early endosomes, as well as promoting the fusion between early endosomes (Hoffenberg et al., 2000; Woodman, 2000). RAB7 is a late endosome/lysosome-associated small GTPase that participates in crucial endocytic processes (Langemeyer et al., 2018). It is involved in several regulatory mechanisms linked to sorting of endosomes, biogenesis of lysosome, and in the formation of phagosomes, autophagosomes, and other lysosome-associated organelles (Gutierrez et al., 2004; Guerra and Bucci, 2016). RAB7 modulates lysosomal biogenesis by associating with the RAB-interacting lysosomal protein (RILP) and affects the morphology and spatial distribution of lysosomes by regulating the cytoskeleton (Cantalupo et al., 2001; Wang et al., 2004; Figure 3).

Notably, multiple RAB proteins are involved in the various stages of autophagic activity. RAB1, RAB4, and RAB11 regulate membrane trafficking originating in different organelles and direct them to the phagophore assembly site (Zoppino et al., 2010; Longatti and Tooze, 2012; Talaber et al., 2014). While, RAB5 interactions with the BECN1/PI3KC3 complex regulates the nucleation of the phagophore membranes (Morris et al., 2015; Pavlinov et al., 2020). RAB33 associates with Atg16L1, thus enlarging phagophore membrane area in a GTP-dependent manner (Itoh et al., 2008). Lastly, late endosome associated RAB proteins, RAB7, and RAB24, mediate the fusion of autophagosomes with lysosomes, and the clearance of autophagic compartments during basal conditions (Gutierrez et al., 2004; Ylä-Anttila et al., 2015; Ylä-Anttila and Eskelinen, 2018; Li et al., 2023). Importantly, a link between synaptic vesicle recycling and autophagy has been demonstrated *via* the interaction of synaptic vesicle-associated RAB26 with phagophore elongation factor Atg16L1. This leads to the targeting of synaptic vesicles to phagophores for bulk degradation (Binotti et al., 2015; Lüningschrör et al., 2017).

Differentially Expressed in Normal and Neoplastic cells-containing proteins primarily function as GEFs or GAPs for RAB GTPases (Yoshimura et al., 2010; Marat et al., 2011; Wu et al., 2011). They can also function as ADP-ribosylation factors (ARFs) (Su et al., 2020), and as Ras-related GTP binding proteins (RRAGs) (Müller and Goody, 2018; Lawrence et al., 2019). As C9ORF72 also belongs to the DENN family of proteins, it is likely that C9ORF72 serves as a GEF or GAP for multiple RAB GTPases (Levine et al., 2013a; Tang et al., 2020). Moreover, C9ORF72 not only interacts with several RAB molecules and key modulators of membrane trafficking (Tang, 2016; Boivin et al., 2020), but also because of its complex and multifaceted interactions, C9ORF2 protein harbors the potential to regulate autophagy at multiple steps. The identification of an unexpected interaction between C9ORF72 and RAB1A, which is involved in autophagy initiation, led to the discovery of a novel function for C9ORF72 protein, as an adaptor protein between RAB1A and the ULK1 complex. This interaction enables the translocation of the ULK1 complex to predetermined sites of autophagosome formation and links C9ORF72 in initial steps of autophagy (Webster et al., 2016). RAB5A is an important determinant in the process of autophagy initiation, and is mainly involved in delivering PtdIns3K complex and ATG7/ATG5/LC3 lipid complex to the newly formed phagophore (Li et al., 2013; Lin et al., 2016). An interaction between RAB5 and C9ORF72 has been identified in human motor neurons (Farg et al., 2017; Shi et al., 2018). Notably, a significant reduction in autophagosome numbers due to deficits in autophagosome formation was observed in *C9ORF72*-ALS patient-derived motor neurons (Shi et al., 2018). The elimination of *C9ORF72* haploinsufficiency *via* the over expression of C9ORF72 protein, or the expression of a constitutively active RAB5A construct, could rescue the autophagosome formation deficits and enhance the survival of C9ORF72 neurons. These experiments convincingly implicate a critical role for this interaction in the cell clearance machinery as well as causally linking autophagosome formation deficits in the pathogenesis of *C9ORF72*-ALS-FTD (Shi et al., 2018).

Importantly, the C9ORF72-SMCR8-WDR41 complex confers GEF activity on RAB8A and RAB39B as well as GAP activity on RAB8A and RAB11A, thus playing a role in the activation of autophagy, and controlling autophagic flux (Yang et al., 2016). Since, RAB8A and RAB39B interact with other ALS-associated proteins such as TBK1, OPTN, and SQSTM1/p62, this suggests that autophagy dysfunctions are linked with the ALS pathogenesis (Corbier and Sellier, 2017). As expected, the knockdown of *C9ORF72* in neurons led to impaired autophagy and concomitant accumulation of TARDBP and SQSTM1/p62 aggregates, recapitulating histopathological hallmarks of ALS-FTD (Sellier et al., 2016). It is now established that TBK1, SMCR8, and the C9ORF72 complex together with RAB39B belong to a common pathway regulating autophagy. This finding was inferred from experiments showing that TBK1 phosphorylates SMCR8, and the reduction in TBK1 expression levels can be normalized either by employing phosphomimetic mutants of SMCR8 or by over expressing the constitutively active version of RAB39B (Sellier et al., 2016). An additional role of the complex can be deduced from studies showing that the C9ORF72 complex regulates ARF GTPases and RAG GTPases. This regulation is mainly through SMCR8 which possesses GAP activity toward ARF1 and ARF6 GTPases, thereby regulating endocytic and Golgi trafficking (Sivadasan et al., 2016; Wang M. et al., 2020; Su et al., 2021). RAB8A can actively recruit TBK1, a protein kinase, which is then involved in the dual regulation of antiviral innate immunity and autophagy. Although TBK1 does not exhibit any kinase activity for C9ORF72, TBK1 phosphorylates SMCR8, ULK1, OPTN, and SQSTM1/p62. This in turn promotes the interaction with LC3, thereby facilitating the autophagic clearance of pathogenic proteins (Freischmidt et al., 2015; Heo et al., 2015; Yang et al., 2016; Harding et al., 2021). Recently, it was identified that TBK1 is hyperphosphorylated and sequestered within PolyGA aggregates, leading to the loss of TBK1 activity, enhancing TARDBP aggregation and triggering neurodegeneration in *C9ORF72* ALS-FTD (Shao et al., 2022). See Table 1 describing the involvement of RAB proteins in ALP and the association of C9ORF72 proteins with specific RAB proteins. *C9ORF72* haploinsufficiency leads to dysfunctional ALP, which accelerates the accumulation of toxic DPRs (Zhu et al., 2020; Beckers et al., 2021). Notably, DPRs such as PolyGA compromise neuronal proteostasis by accumulating within 26S proteasome and stalling cellular degradation (Guo et al., 2018). These observations indicate that, within the context of *C9ORF72* ALS-FTD, the cross talk between ALP and UPS might be impaired, and that targeting both processes simultaneously could be a beneficial approach in treating *C9ORF72* ALS-FTD.

**TABLE 1:** List of RAB proteins involved in the autosomal-lysosomal pathway (ALP).

RAB	Autophagy stage	C9ORF72 interaction	References
RAB1A	Autophagy activation and induction	+	Webster et al., 2016
RAB39A		Unknown	Niu et al., 2020
RAB24		Unknown	Munafó and Colombo, 2002
RAB37	Initiation of pre-autophagosome formation	Unknown	Sheng et al., 2017
RAB7	Lysosome reformation during autophagy	+	Yu et al., 2010; Farg et al., 2017
RAB1A	Autophagosome formation	+	Carlos Martín Zoppino et al., 2010; Hutagalung and Novick, 2011; Farg et al., 2017
RAB1B		+	Winslow et al., 2010; Hutagalung and Novick, 2011; Farg et al., 2017
RAB4		Unknown	Hutagalung and Novick, 2011; Talaber et al., 2014
RAB5		+	Ravikumar et al., 2008; Hutagalung and Novick, 2011; Farg et al., 2017
RAB7		+	Yamaguchi et al., 2009; Hutagalung and Novick, 2011; Farg et al., 2017
RAB9A		Unknown	Hutagalung and Novick, 2011; Nozawa et al., 2012; Yan et al., 2013
RAB10		Unknown	Limanaqi et al., 2018
RAB11		+	Fader et al., 2007; Morvan et al., 2009; Hutagalung and Novick, 2011
RAB32		Unknown	Hirota and Tanaka, 2009; Hutagalung and Novick, 2011; Wang C. et al., 2012
RAB33B			Itoh et al., 2008; Hutagalung and Novick, 2011
RAB7	Autophagosome maturation	+	Hutagalung and Novick, 2011; Hyttinen et al., 2013; Farg et al., 2017
RAB8B		+	Hutagalung and Novick, 2011; Pilli et al., 2012; Sellier et al., 2016
RAB24		Unknown	Ao et al., 2014
RAB9		Unknown	Nozawa et al., 2012
RAB10		Unknown	Limanaqi et al., 2018
RAB11		+	Fader et al., 2007; Farg et al., 2017
RAB33B		Unknown	Itoh et al., 2011
RAB39B		+	Pilli et al., 2012; Sellier et al., 2016
RAB8A	PINK-dependent mitophagy	+	Lai et al., 2015; Sullivan et al., 2016
RAB8B		+	Ginns et al., 2014
RAB13		Unknown	Lai et al., 2015
RAB5	Links SVs to pre-autophagosomal structures	Unknown	Limanaqi et al., 2018
RAB26		Unknown	Binotti et al., 2015
RAB35		+	Wu et al., 2011; Limanaqi et al., 2018

Further, the direct interaction of C9ORF72 with various RAB proteins are indicated. Note the interaction/involvement of C9ORF72 with RAB proteins at multiple levels of the ALP, which are indicated by a (+) sign. SVs, synaptic vesicles.

In the context of mTORC1 signaling, a reduction in the phosphorylation of RPS6KB1/p70S6K has been established in C9ORF72 knockdown models, implicating reduced mTORC1 activation (Wang M. et al., 2020). Any reduction in mTORC1 signaling leads to the nuclear translocation of transcription factors TFEB and TFE3. This translocation enhances transcription of genes associated with autophagy and lysosomal pathways, thus causing an excessive autophagic flux (Tang, 2016; Ji et al., 2017). Overall, loss of *C9ORF72* function can synergistically exert dual responses on the regulation of autophagy. Firstly, by reducing mTORC1 signaling, thereby over-activating autophagy. Secondly, by reduced interaction with RAB GTPases due to *C9ORF72* haploinsufficiency, thus causing impairment in vesicular trafficking. This impairment in trafficking hinders the proper functioning of the endosomal lysosomal pathways.

## Physiological and pathological roles of C9ORF72 in lysosomal pathways

It is well established that deficits in lysosomal function and lysosomal biogenesis can invariably affect autophagic turnover and enhance cellular accumulation of autophagosomes. Considering that C9ORF72 has been reported to display GEF activity toward GTPases, a novel interaction of C9ORF72 *via* its DENN domain with inactive RRAG GTPases was identified, suggesting a novel role for C9ORF72 in nutrient sensing and lysosomal biogenesis (Martina and Puertollano, 2013; Corbier and Sellier, 2017; Fukuda and Shiozaki, 2018). During episodes of cellular starvation, the inactivation of mTORC1, results in the loss of the inhibitory effect of mTORC1 on ULK1. This causes the release of the ULK1 complex and the recruitment of the C9ORF72 complex to the lysosomes by SLC66A1. The movement of C9ORF72 to the lysosome is mediated *via* the interaction between SLC66A1 and WDR41 (Amick and Ferguson, 2017). Notably, an accumulation of enlarged lysosomes together with elevated expression of SQSTM1/p62 and LC3 has been observed in rodent C9ORF72 knock out macrophages, microglia and patient-derived motor neurons, suggestive of an increase in autophagy and/or a deficit in the clearance of autophagosomes by lysosomes (O’Rourke et al., 2016; Sullivan et al., 2016; Ugolino et al., 2016). Moreover, mice lacking C9ORF72 also exhibited an increased number and size of lysosomes within neurons, indicating increased lysosomal biogenesis as well as the accumulation of dysfunctional lysosomes (Shao et al., 2020). The well characterized accumulation of enlarged autophagosomes in *C9ORF72* loss of function models has now been linked to M6PR vesicle trafficking (Saftig and Klumperman, 2009; Banik et al., 2020; Beckers et al., 2021). These vesicles bud from the *trans*-Golgi network and are actively involved in the transport of M6P-tagged lysosomal proteins to endosomes, thereby sustaining lysosomal biogenesis (Saftig and Klumperman, 2009). Intriguingly, in *C9ORF72* knock out cells, M6PR is mislocalized within the cytoplasm in comparison to its correct perinuclear localization, suggesting that altered M6PR function within cells accounts for the observed changes in lysosomal biogenesis.

C9ORF72 and SMCR8 have been shown to play a role in regulating lysosomal exocytosis, involving the fusion of the lysosome with the plasma membrane, followed by the release of lysosomal contents (Medina et al., 2011). Likewise, the level of LAMP1 is increased in C9ORF72 or SMCR8-deficient macrophages, suggesting an increase in lysosomal exocytosis (Zhang et al., 2018; Shao et al., 2020). This enhanced lysosomal exocytosis is further consolidated by the presence of elevated extracellular levels of lysosome enzymes: cathepsin B and cathepsin D in *C9ORF72-*deficient cells (Shao et al., 2020). Moreover, C9ORF72 is implicated in the process of lysosomal targeting of CARM1, a histone arginine methyltransferase known to work as an epigenetic regulator of autophagy. During conditions of starvation, C9ORF72 interacts with CARM1, and facilitates its transport to the lysosome for degradation. C9ORF72 deficiency or reduction leads to cytoplasmic accumulation of CARM1, which translocates to the nucleus and functions as a transcriptional co-activator of autophagic and lysosomal genes such as Atg14 and Map1lc3b. CARM1 has been shown to be highly enriched on the promoters regulating autophagy and lysosomal genes in *C9ORF72*-deficient cells. This observation suggests that *C9ORF72* haploinsufficiency leads to the dysregulated expression of genes involved in autophagy and lysosomal pathways (Liu and Wang, 2019). Importantly, several studies indicate a role for C9ORF72 in nutrient-sensing within the lysosome, independent of mTORC1 signaling (Ugolino et al., 2016; Amick et al., 2020; Pang and Hu, 2021). Thus, the recruitment of the C9ORF72-SMCR8-WDR41 complex to the lysosomes might be an active component of the lysosome-associated pathway, regulating the cellular response to nutrient variation (Amick et al., 2018). Additionally, the recruitment of C9ORF72 complex to the lysosome also inadvertently facilitates the interplay with mTORC1 (Wang M. et al., 2020). Notably, these two processes involving the C9ORF72-related network may reflect functions that parallel each other.

## Therapeutic strategies for targeting ALP in *C9ORF72*-ALS-FTD

The lack of preventive or curative therapy for ALS-FTD, makes the targeting of ALP an attractive option. Given the strong evidence for disrupted ALP in all major forms of NDs, therapeutic strategies aimed at restoring autophagy deficits represent a common hub that would be beneficial to all NDs. One likely direction involves the early activation of autophagy, thereby enhancing the clearance of aggregated proteins. Notably, the role of rapamycin, one of the most well-known autophagy activators, is still debatable as it acts on multiple systems including the immune system. Different studies have reported both neuroprotective and detrimental effects when tested *in vivo* on various ALS animal models (Zhang et al., 2011; Wang I et al., 2012). Several studies have employed small-molecule autophagy activators such as trehalose, lithium, berberine, promethazine, and carbamazepine to achieve neuroprotection in ALS-FTD (Boivin et al., 2020; Lu et al., 2022). Other well-known autophagy-activating compounds, such as lithium, valproic acid, or carbamazepine, are already being employed in the clinics due to their positive effect on mood-stabilization. This class of compounds mainly act on inositol triphosphate (IP3) metabolism *via* the inhibition of PI3K and GSK-3β. IP3 acts on its receptor IP3R located at the ER thus promoting calcium release and ATP production that counteracts autophagic flux. Therefore the usage of mood stabilizing drugs, such as lithium, depletes IP3 viability and thus effectively boosts the autophagy process (Sarkar et al., 2005; Harris and Rubinsztein, 2012).

Furthermore, histone deacetylase 6 (HDAC6) has a relevant role in the process of fusion between autophagosomes and lysosomes and its therapeutical effect has been tested in animal models of superoxide dismutase 1 (SOD1)-linked ALS, where its genetic inhibition prolonged the lifespan of the animals (Taes et al., 2013). HDAC6 inhibitory effect in ameliorating ALS-FTD pathology has been tested in C9ORF72 mutation, where its inhibition reduced the accumulation of PolyGA *in vivo* (del Rosso et al., 2022). Interestingly, Metformin, an anti-hypertensive drug has been found to induce autophagy *via* both AMPK-dependent and SIRT1-dependent pathways and was employed in alleviating C9ORF72-linked DPR pathology in *C9-500* BAC mouse model (Song et al., 2015; Zu et al., 2020). As Polito et al. (2014) have demonstrated, the overexpression of TFEB in AD rodent models inhibited neurofibrillary tangle pathology and improved neuronal survival and function. Yet another approach would be to enhance nuclear TFEB levels by reducing its nuclear export by selective inhibitors of nuclear export (SINEs) (McDermott, 2019). Several compounds that regulate the nuclear translocation of TFEB, such as 3,4-dimethoxychalcone (3,4-DC), (Chen et al., 2019) 2-Hydroxypropyl-β-cyclodextrin (HPβCD), (Song et al., 2014), Digoxin, proscillaridin A, and digoxigenin (Wang et al., 2017) have been employed. However, most of these compounds also modulate calcium signaling or mTORC1 or AKT, or PKC signaling pathways, thus it remains unclear whether the effects are directly TFEB specific (McMillan and Dwyer, 1989; Tong and Song, 2015).

**FIGURE 4:**
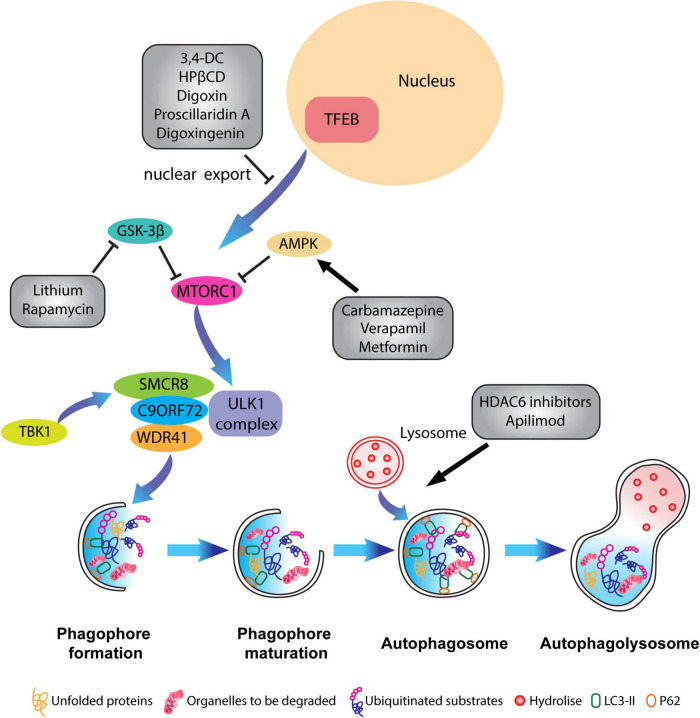
Pharmacological targeting of the autophagy pathway in neurons. Schematic representation of different compounds that have been used to alleviate ALP impairments and to restore autophagic flux. Some therapeutical agents promote the activation or inhibition of specific kinases which block mTORC1 activity such as Carbamazepine, Verapamil and Metformin, or inhibit the translocation of TFEB from the nucleus to the cytoplasm like in the case of 3,4-dimethoxychalcone (3,4-DC), 2-Hydroxypropyl-β-cyclodextrin (HPβCD), Digoxin, proscillaridin A. Other molecules such as HADC6 inhibitors and apilimod promote the fusion of lysosomes with the autophagosome.

Another approach would be to reduce DPRs burden within motor neurons. One method involves the overexpression of heat shock protein B8 (HSPB8), which led to the increase in the autophagy-mediated degradation of DPRs, and enabled the clearance of aggregation prone proteins before they could convert into inclusions (Liu et al., 2022). Furthermore, an interesting natural compound called berberine has been shown to influence TDP-43 protein aggregations boosting its degradation *via* autophagy (Chang et al., 2016). It is crucial to take into consideration the precise stage of the autophagy pathway on which the active compounds and drugs are acting. It has been reported that different active molecules like tamoxifen, progesterone, bosutinib, and lithium all exhibited beneficial effect in ameliorating ALS disease progression *in vivo*, because of increased autophagic flux (Figure 4). Nevertheless, other molecules (i.e., rapamycin and pimozide), despite increasing autophagy concomitantly, suppressed autophagy-mediated degradation. This resulted in the accumulation of autophagosomes and autophagy-related substrates such as misfolded protein aggregates together with SQSTM1/p62 (Fornai et al., 2008; Zhang et al., 2011; Wang I et al., 2012; Kim et al., 2013; Pozzi et al., 2018). Several studies have established a beneficial role for the overexpression of Parkin on α-synuclein toxicity (Shimura et al., 2001; Petrucelli et al., 2002; Rana et al., 2013; Meng et al., 2020). Notably, Parkin prevented the accumulation of SOD1 aggregates in ALS. In particular, Parkin fostered the Lys63-linked polyubiquitination of misfolded SOD1, promoting the clearance of aggregation-prone SOD1 through elevated autophagy (Yung et al., 2016). Given the beneficial effect of Parkin, several small molecules serving as activators of Parkin have been discovered which could be therapeutically employed for the clearance of toxic aggregates *via* autophagy (Miller and Muqit, 2019; Clark et al., 2021).

Lastly, within the context of *C9ORF72*-ALS-FTD, targeting RAB GTPases could serve as a potential therapeutic strategy, as RAB GTPases are extensively involved in the biogenesis and function of endosomal-lysosomal systems (Langemeyer et al., 2018). PIKFYVE is a kinase that converts PtdIns3P to PtdIns (3, 5) P2, thereby inhibiting the fusion between autophagosomes and lysosomes. Inhibition of PIKFYVE kinase by Apilimod counteracts and promotes the fusion of lysosomes and autophagosomes, thereby normalizing lysosomal and autophagosomal deficits in *C9ORF72* patient-derived motor neurons (Shi et al., 2018, 2019). RAB7 receptor antagonist CID1067700 inhibits the excessive transportation of cathepsin B from late endosomes to lysosomes, sustaining lysosomal membrane permeability and thereby reducing reactive astrogliosis in brain injury models (Qin et al., 2019). Although, lack of specificity toward RABs has been the biggest hinderance in the development of RAB-selective antagonists, viral-mediated expression of defined dominant negative/constitutively active RABs might enable the restoration of dysfunctional endosomal-lysosomal pathways.

## Conclusion

It is important to understand the precise steps and function of ALP before attempting to implement it as a therapeutic procedure that potentially may lead to breakthroughs in tackling NDs. In the case of *C9ORF72*-ALS-FTD, the complex and multi-layered function of the protein in regulating ALP as well as endosomal trafficking has just begun to emerge, and further studies are required to dissect its precise function. The presence of *C9ORF72* haploinsufficiency coupled together with the gain of function mechanisms involving both toxic DPRs and HRE further complicates the understanding of these events in the pathophysiology of *C9ORF72*-ALS-FTD. Lastly, the detailed understanding of processes such as phosphorylation, which regulates the initiation as well as the completion of the ALP, is required for future modulation of autophagy. Despite, this tangled scenario, it has become well established that ALP signaling is disrupted in NDs and especially in *C9ORF72*-ALS-FTD, and thus modulating or enhancing ALP might exert a beneficial outcome on the disease pathogenesis. Lastly such approaches might serve as an attractive entry point for future druggable targets.

## Author contributions

SS wrote the review with equal contributions from FP and RD. FP designed all figures. All authors read and approved the submitted version.
