# Erratum: Autophagy and neurodegeneration: unraveling the role of C9ORF72 in the regulation of autophagy and its relationship to ALS-FTD pathology

**DOI:** 10.3389/fncel.2023.1225439

**Published:** 2023-06-07

**Authors:** Frontiers Production Office

**Affiliations:** Frontiers Media SA, Lausanne, Switzerland

**Keywords:** C9ORF72, ALS-FTD, autophagy, autophagy-lysosomal pathway, RAB proteins, endosomal trafficking, neurodegeneration

Due to a production error, the contributions of authors Rim Diab and Federica Pilotto were not included in the author contributions in the published article. The corrected Author Contributions Statement appears below.

“SS wrote the review with equal contributions from FP and RD. FP designed all figures. All authors read and approved the submitted version.”

The publisher apologizes for this mistake. The original article has been updated.

